# A disulphide isomerase gene (*PDI-V*) from *Haynaldia villosa* contributes to powdery mildew resistance in common wheat

**DOI:** 10.1038/srep24227

**Published:** 2016-04-13

**Authors:** Muhammad Faheem, Yingbo Li, Muhammad Arshad, Cheng Jiangyue, Zhao Jia, Zongkuan Wang, Jin Xiao, Haiyan Wang, Aizhong Cao, Liping Xing, Feifei Yu, Ruiqi Zhang, Qi Xie, Xiue Wang

**Affiliations:** 1 The State Key Laboratory of Crop Genetics and Germplasm Enhancement, Cytogenetics Institute, Nanjing Agricultural University/JCIC-MCP, Nanjing, Jiangsu 210095, P.R. China; 2 Biotech Research Institute, Shanghai Academy of Agricultural Sciences, Shanghai Key Laboratory of Agricultural Genetics and Breeding, Shanghai 201106, China; 3 Institute of Genetics and Developmental Biology, Chinese Academy of Sciences, Beijing 100101, China

## Abstract

In this study, we report the contribution of a *PDI-like* gene from wheat wild relative *Haynaldia villosa* in combating powdery mildew. PDI-V protein contains two conserved thioredoxin (TRX) active domains (a and a′) and an inactive domain (b). PDI-V interacted with E3 ligase CMPG1-V protein, which is a positive regulator of powdery mildew response. PDI-V was mono-ubiquitinated by CMPG1-V without degradation being detected. *PDI-V* was located on *H. villosa* chromosome 5V and encoded for a protein located in the endoplasmic reticulum. *Bgt* infection in leaves of *H. villosa* induced *PDI-V* expression. Virus induced gene silencing of *PDIs* in a *T. durum-H. villosa* amphiploid compromised the resistance. Single cell transient over-expression of *PDI-V* or a truncated version containing the active TXR domain a decreased the haustorial index in moderately susceptible wheat cultivar Yangmai 158. Stable transgenic lines over-expressing *PDI-V* in Yangmai 158 displayed improved powdery mildew resistance at both the seedling and adult stages. By contrast over-expression of point-mutated *PDI-V*^*C57A*^ did not increase the level of resistance in Yangmai 158. The above results indicate a pivotal role of *PDI-V* in powdery mildew resistance and showed that conserved TRX domain a is critical for its function.

Every year plant diseases are responsible for billions of dollars worth of crop losses worldwide. A comprehensive understanding of how plants coordinate their defense systems will facilitate exploitation of the most effective protection strategies. Powdery mildew, caused by the fungus *Blumeria graminis* (DC) f. sp. *tritici* (*Bgt*), is one of the most important foliar diseases of wheat worldwide^1^. Yield losses caused by the disease may be as high as 50%^2^. Plants possess remarkably sophisticated mechanisms to fend off attack by pathogens and pests. The first line of defense by the plant immune system against pathogens involves basal defense responses that are triggered upon detection of pathogen associated molecular patterns (PAMPs)^3^,^4^. To suppress PAMP-triggered immunity (PTI) pathogens secrete small unique protein molecules known as effectors. Upon recognition, host plants trigger a second line of defense, effector triggered immunity (ETI)^5^,^6^.

The identification of genes related to non-host resistance and regulators of PTI will help breeders to access durable resistance. Powdery mildew resistance gene *Pm21* from *Haynaldia villosa* (Syn. *Dasypyrum villosum*, 2n = 2x = 14, genome VV), confers durable broad-spectrum resistance (BSR) to *Bgt*^7^. The *Bgt*-*H. villosa* provides a useful pathosystem for elucidating the mechanism of BSR. In a previous study the resistance pathway was analyzed by comparing transcription patterns of *H. villosa* before and after *Bgt* infection by microarray analysis using the Barley1 Genechip (Affymetrix). Based on the microarray results two genes, a serine/threonine kinase gene *Stpk-V* and an E3 ligase gene *CMPG1-V* were cloned and both positively contributed to BSR when over-expressed in wheat^1^,^2^. Further clarification of resistance signaling pathways and identification of more genes related to disease response will facilitate an understanding of the mechanism of *Pm21*-mediated BSR.

The ubiquitin proteasome system (UPS) is a highly regulated mechanism of intracellular protein degradation in eukaryotes. Many studies over the last decade, have shown that E3 ligase genes are involved in regulation of innate immunity in plants^8^,^9^. The functions of a dozen E3 ligase genes in plant immunity were recently well characterized in *Arabidopsis*^10^. An *in vitro* ubiquitination activity study showed that CMPG1-V is a positive regulator of the powdery mildew resistance. CMPG1-V-mediated resistance involves ROS and SA pathways^1^. To further elucidate the molecular pathway of CMPG-V-mediated powdery mildew resistance, a yeast two hybrid cDNA library of *H. villosa* leaves was constructed and screened for interacting cDNA-expressed genes. One of the positive cDNA clones encoded a disulphide isomerase (PDI)-like protein.

PDI (EC 5.3.4.1), a ubiquitious sulfydryl oxidoreductase found in abundance in the lumen of the endoplasmic reticulum (ER) of all eukaryotic cells, is an important cellular protein with multiple biological functions, displays versatile redox behavior, is highly interactive with other proteins and has an implied role in various diseases^11^,^12^. The fundamental role of PDI-like proteins and their wide range of substrates make it very difficult to understand their function *in vivo*, especially in plants^13^,^14^. However, specific functions of plant PDIs have been postulated and supported with accumulating evidence. *Arabidopsis thaliana* encodes 12 PDI-like proteins. Among them *PDI5* regulates the timing of programmed cell death (PCD) in endothelial cell^15^, while *PDIL-2;1* plays a critical role in the development of the embryo sac^16^. Typical wheat PDIs are involved in the assembly of storage protein within the ER and also participate in quality control by regulation of unfolded proteins^14^,^17^,^18^,^19^,^20^. The barley variant *HvPDIL5-1* confers BSR against many strains of Bymoviruses^21^.

PDI is one of the redox proteins that regulate reactive oxygen species (ROS) production by the Nox enzyme family, as well as changes in the redox status of cells to activate the defense system^22^. Calcium influx and nitric oxide (NO) production results in the S-nitrosylation of PDI (SNO-PDI), which increases the level of polyubiquitinated proteins and triggers cell death^23^ and inhibition of mitochondria, leading to the generation of ROS and NO^24^. Antioxidant properties of PDI could help limit potential cell damage by ROS generated during pathogen infection. PDI may be integral to the repertoire of mechanisms that host plants have evolved to suppress the highly destructive and energy-consuming processes accompanying hypersensitive responses^25^. Plant defense systems produce ROS which not only causes damage to hydrolytic enzymes in pathogens but also regulate various cellular pathways during infection^26^. Some studies in plants have suggested the role of cell surface PDI in transportation of defense-signaling cascades i.e. movement of NO between cells^27^,^28^,^29^. ER-quality control components (which also involve PDI) directly take part in PTI as any disturbance of ER-localized processing of the PAMP receptor EFR1 disrupts PTI response and results in enhanced susceptibility to bacterial and fungal pathogens^30^,^31^.

Only one study in wheat has reported involvement of a *PDI* gene in defense; this was against the fungal pathogen *Mycosphaerella graminicola*^25^. Here, we report the cloning and functional analysis of a *PDI-like* gene from *H. villosa* in combating powdery mildew. *H. villosa*, a wild diploid grass, belongs to the tertiary wheat gene pool and possesses high levels of resistance to abiotic and biotic stresses, including resistance to powdery mildew. The present research aimed to investigate the interaction of PDI-V with CMPG1-V, to examine possible involvement of *PDI-V* during powdery mildew infection using molecular approaches, and to fully characterize the *PDI-V* gene in response to different phytohormone and abiotic stress treatments.

## Results

### *PDI-V* was cloned from *H. villosa* by screening a Y2H cDNA library using CMPG1-V as bait

**Figure 1:**
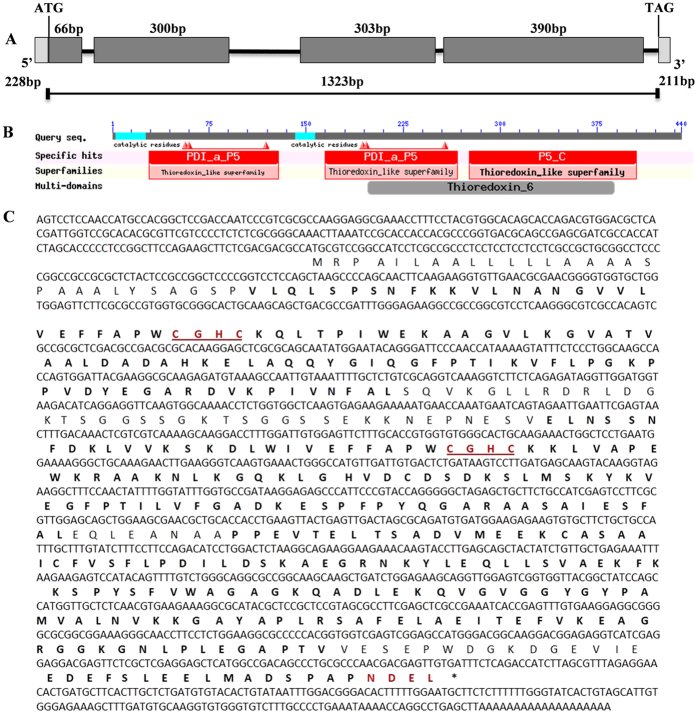
Structural features of the *PDI-V* cDNA sequence and its translation product. (**A**) Structure deduced from the full-length cDNA sequence of PDI-V. The size of each domain, signal peptide and 3′ and 5′- UTR sizes and position of the ATG and TAG stop codons are shown. (**B**) Graphical representation of domain organization as provided by the NCBI conserved domain database. (**C**) Deduced amino acid sequence of PDI-V. Thioredoxin catalytic motifs and ER retention signals are shown in red.

CMPG1-V positively regulates powdery mildew resistance in common wheat^1^. To identify proteins interacting with CMPG1-V, a Y2H cDNA library of *H. villosa* was constructed and used to elucidate the resistance pathway mediated by CMPG1-V^32^. Using CMPG1-V as bait, 17 putative cDNA clones interacting with CMPG1-V were identified (Supplementary Table S1); one of them encoded a protein disulphide isomerase (PDI). Based on the cDNA sequence, the 1,615 bp full-length *PDI* gene was isolated from *H. villosa*. The gene has an ORF of 1,323 bp, encoding a protein with 440 amino acids (Fig. 1A,B). BLASTn analysis showed that the sequence was highly similar to the *T. aestivum* PDI-like genes *TaPDIL5-1a* & *1b*, and hence was designated *PDI-V.*

PDI-V has two tandem thioredoxin active domains (a and a′), each containing a typical tetra peptide motif (CGHC) at the N terminus, and an inactive thioredoxin b domain at the C-terminus. Signal P analysis predicted the presence of a signal peptide from the start of the N terminus to the 30^th^ amino acid, and a modified NDEL signal for retention in the ER at the C-terminus (Fig. 1C). Alignment of PDI-V with homologous proteins from other grass species and *Arabidopsis* indicated that PDIs are well conserved across mono- and dicotyledonous plants (Supplementary Fig. S1). Phylogenetic analysis showed that *PDI-V* homologs from monocots clustered into one clade, and *PDI-V* was closest to *PDIs* from *Hordeum vulgare, T. aestivum, T. urartu* and *Aegilops tauschii* (Supplementary Fig. S2A).

### PDI-V interacted with, and is ubiquitinated, by CMPG1-V without detectable degradation

**Figure 2:**
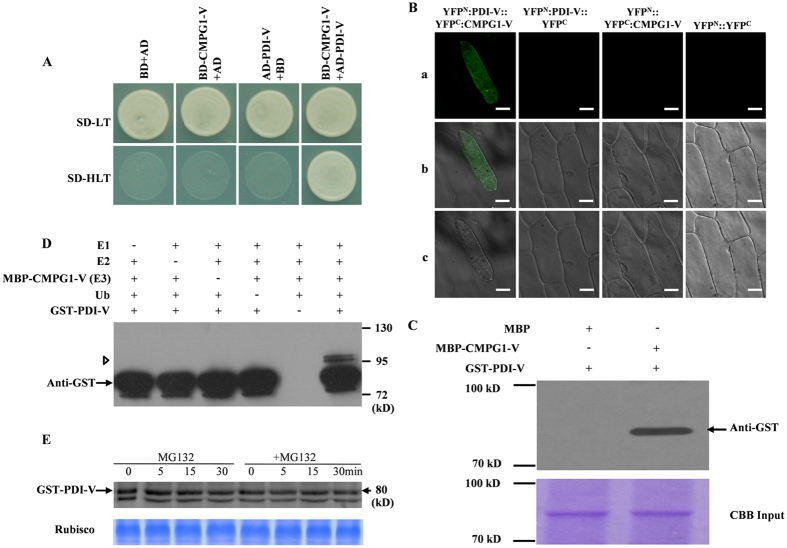
Interaction of PDI-V with CMPG1-V. (**A**) Y2H assay showing interaction of PDI-V and CMPG1-V. Yeast growth on SD-Leu/-Trp/-His confirms protein: protein interaction. (**B**) *In vivo* interaction of YFP^N^:PDI-V with YFP^C^:CMPG1-V in the BiFC assay in onion epidermal cells. Epiflourescence (a) bright field (b) and light images (c) of YFP^N^:PDI-V::YFP^C^:CMPG1-V, YFP^N^:PDI-V, YFP^C^:CMPG1-V and empty vectors YFP^N^:YFP^C^ (**C**) *In vitro* interaction of MBP-CMPG1-V with GST-PDI-V by pull-down assay. GST protein loading is shown by Coomassie brilliant (CBB) blue staining (bottom panel). The position and size of the molecular marker are shown to the left of the blot (**D**) *In vitro* ubiquitination assay of recombinant GST-PDI-V fusion protein by MBP-CMPG1-V. The recombinant protein was assayed for E3 activity in the presence of E1, E2, biotinylated ubiquitin (Ub) and GST-PDI-V. + and – represent the presence and the absence of the corresponding protein in the reaction mixture respectively. The position and size of the molecular marker are shown to the right of the blot. (**E**) Cell-free degradation assay of recombinant GST-PDI-V. Total protein extracted from wheat leaves was incubated with MBP-CMPG1 and GST-PDI-V at 30 °C for 90 min with or without MG132, with sampling at different time points. GST-PDI-V protein was detected by western blotting using anti-GST antibody.

Y2H confirmed the interaction of PDI-V with CMPG1-V in yeast (Fig. 2A). To test their physical interaction *in vivo*, bimolecular fluorescence complementation (BiFC) with split yellow fluorescent protein (YFP) was employed. Co-expression of nYFP-PDI-V and cYFP-CMPG1-V in onion epidermal cells resulted in complementation of YFP in the cytoplasm, proving their interaction *in vivo* (Fig. 2B).

To further confirm the physical interaction revealed by Y2H and BiFC assays, a pull-down assay was performed. A specific 80 kDa band for GST-PDI-V was obtained only when using MBP-CMPG1-V (Fig. 2C, Lane 2), but not when using MBP protein alone (Fig. 2C, Lane 1), further confirming the existence of physical interaction between CMPG1-V and PDI-V. CMPG1-V acts as an U-box/ARM E3 ubiquitin ligase. Ubiquitination of PDI-V by CMPG1-V was demonstrated by an *in vitro* ubiquitination assay. In the presence of all requisite components for ubiquitination, GST-PDI-V was monoubiquitinated by MBP-CMPG1-V, as evidenced by increased size of the target band (Fig. 2D, Lane 6). A cell-free degradation assay showed that, regardless of the presence or absence of 26S proteasome inhibitor reagent MG132, the amount of GST-tagged PDI-V showed no significant change when co-incubated with MBP-tagged CMPG1-V, indicating GST-tagged PDI-V could not be degraded by CMPG1-V and hence should not be its degradation target (Fig. 1E).

### *PDI-V* gene is located on long arm of chromosome 5V of *H. villosa*, and PDI-V subcelluarly localized in the ER

A specific primer pair (PDIV-40F/R, Supplementary Table S2) was designed based on alignment of genomic sequences of *PDI-V* and its wheat genome A, B and D homologs. PCR using *H. villosa* (VV), *T. durum-H. villosa* amphiploid (AABBVV), a complete set of Chinese Spring-*H. villosa* addition lines (DA1V-7V) and Chinese Spring as templates showed that the 600 bp band specific for *PDI-V* was amplified only from *H. villosa*, AABBVV and DA5V (Supplementary Fig. S2B), indicating the location of *PDI-V* on chromosome 5V. Further amplification using translocation lines CINAU-61 and CINAU-158 showed that the *PDI-V* band was amplified only by CINAU-158 (Supplementary Fig. S2B), indicating the *PDI-V* was located on 5VL. All identified *PDI* orthologs from the sub-genomes of wheat (Chinese Spring A, B and D) and related species barley, rice and Brachypodium were located in syntenic regions of group 5L chromosomes (Supplementary Table S3).

**Figure 3:**
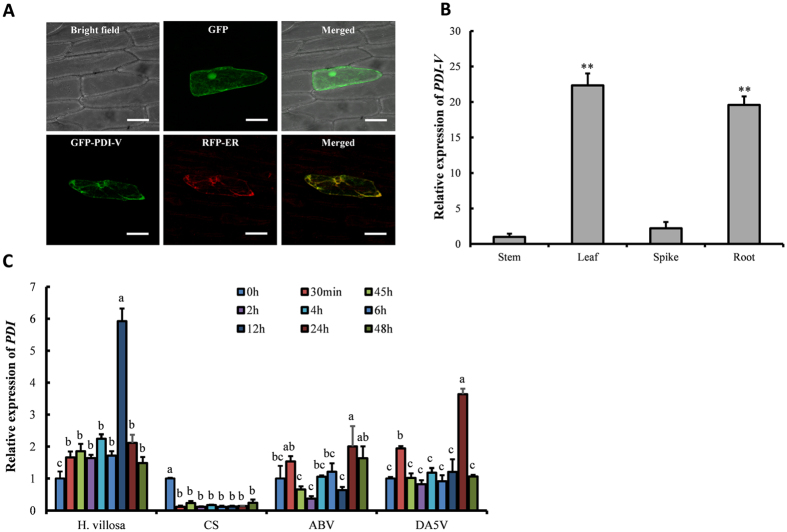
Subcellular localization of PDI-V and expression profiling of *PDI-V*. (**A**) Subcellular localization of GFP-PDI-V co-expressed with RFP in onion epidermal cells. Localization of green fluorescence protein (GFP) as control is shown in the upper panel. The first image in the lower panel shows the green GFP-PDI-V signal localized in the endoplasmic reticulum and the second image shows localization of the RFP ER marker. The third image in this panel shows the co-localization of GFP-PDI-V and RFP-ER. Scale bar = 100 μm. (**B**) Expression pattern of *PDI-V* in tissues (stem, leaf, immature spike and root) of *H. villosa* (**C**) Expression pattern of *PDI* in *H. villosa* and wheat lines i.e. Chinese Spring (CS), *T. turgidum-H. villosa* amphiploid (AABBVV), and the 5V addition line (DA5V) after inoculation with *Bgt*. Each result is the mean of three independent biological repeats while bars show standard deviations. The *Tubulin* gene was used as an internal control to normalize qRT-PCR values. The lower script alphabet letters represent the statistically significant differences at p = 0.05 (one way ANOVA test).

Vectors of PDI-V-GFP and endoplasmic reticulum (ER) marker gene (RFP) were transiently co-expressed in onion epidermal cells to detect subcellular location. GFP-tagged PDI-V protein signals were localized in the ER (Fig. 3A). A complete overlap of GFP and RFP signals was observed in transiently over-expressing cells, confirming that PDI-V-GFP was localized to the lumens of endoplasmic reticulum (Fig. 3A).

### PDI-V plays a positive role in powdery mildew resistance of wheat and domain a is involved in restricting *Bgt* haustorial formation

Quantitative reverse transcription PCR (qRT-PCR) analysis in different tissues of *H. villosa* showed that expression of *PDI-V* was 19- and 22-fold higher in roots and leaves than in stems. *PDI-V* expression in spikes was lower than in leaves and roots, but slightly higher than in stems (Fig. 3B). When two-week-old *H. villosa* seedlings were inoculated with *Bgt*, *PDI-V* expression in leaves increased slightly at 0.5 hours post inoculation (hpi), and reached a 6-fold level compared to the control at 24 hpi (Fig. 3C). In Chinese Spring *PDI* remained at constant levels after inoculation, whereas expression in the AABBVV amphiploid and DA5V increased at 0.5 hpi, but no obvious change was observed from 1 hpi to 12 hpi. At 24 hpi expression in the amphiploid and DA5V was twice that at 0 hpi, but expression levels for both lines were significantly lower than for *H. villosa*. At 48 hpi the *PDI-V* expression remained high in the amphiploid, but had fallen to a pre-treatment level in DA5V (Fig. 3C).

**Figure 4:**
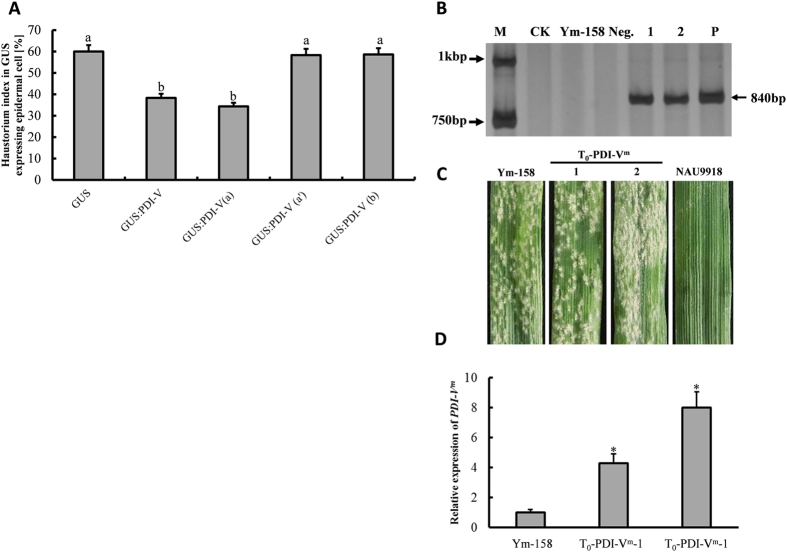
Gene function analysis of *PDI-V*. (**A**) Functional analysis of *PDI-V* by a single cell transient expression assay in wheat leaves by the particle bombardment method. As a control treatment, the GUS-reporting empty vector pWMB002 was transformed into the epidermal layer of susceptible variety Yangmai 158. To assess the function of PDI-V and its truncated domains a, a′ and b, GUS was co-transformed with each construct. The experiment was repeated in three biological repeats and mean data with standard deviations are displayed. Lowercase letters indicate statistically significance differences at p = 0.05 (one way ANOVA). (**B**) PCR of positive point mutated (*PDI-V*^*m*^) T_0_ transgenic plants. M, DNA marker DL2kb; CK, ddH_2_O; Ym-158, Yangmai 158; Neg., negative T_2_ transgenic plant 1 & 2, T0-PDI-V^m^-1 & T0-PDI-V^m^-2 plants; P, plasmid of *pBI220:PDI-V*. (**C**) Interaction of *Bgt* with T_0_-PDI-V^m^ evaluated at 7 dpi using inoculated detached leaves of Yangmai 158 and Nannong-9918 (NAU 9918) as susceptible and resistant controls (**D**) Over-expression of *PDI-V*^*m*^ in T_0_-transgenic plants assayed by qRT-PCR using *Tubulin* as an internal control to normalize the quantification data. Significance was determined by paired sample t-tests. *p < 0.05.

To further characterize the role of *PDI-V* in powdery mildew response, a single-cell transient over-expression assay (TOA) was conducted using the moderate susceptible wheat cultivar (cv) Yangmai 158 as receptor. The haustorial index (HI) for Yangmai 158 was 59.9% when transformed with *GUS* alone, but was significantly decreased to 38.3% when co-transformed with *GUS* and *pBI220-PDI-V* (Fig. 4A). This demonstrated an involvement of *PDI-V* in response to *Bgt*. To determine the PDI-V domain involved three vectors, *viz pBI220-PDI-V*^*a*^, *pBI220-PDI-V*^*a*^′ and *pBI220-PDI-V*^*c*^, were constructed and used in single-cell TOA. Only the domain a construct significantly reduced the HI (34.36%) to a similar level as the full-length *PDI-V* (Fig. 4A). This proved that domain a of PDI-V was involved in restraining the haustorium formation of *Bgt* and restricted their further penetration.

To confirm the essential role of domain *a* in chaperon involvement in *Bgt* response, a point mutation *PDI-V*^*C57A*^ (*PDI-V*^*m*^) was created in the catalytic motif of domain *a*. When *PDI-V*^*m*^ was transformed into Yangmai 158, 13 positive T_0_ transformants overexpressing *PDI-V*^*m*^ were identified (Fig. 4B,D). All showed similar levels of powdery mildew susceptibility to that of non-transformed Yangmai 158 (Fig. 4C), confirming an essential role of domain *a* in function of *PDI-V* in powdery mildew response in wheat.

**Figure 5:**
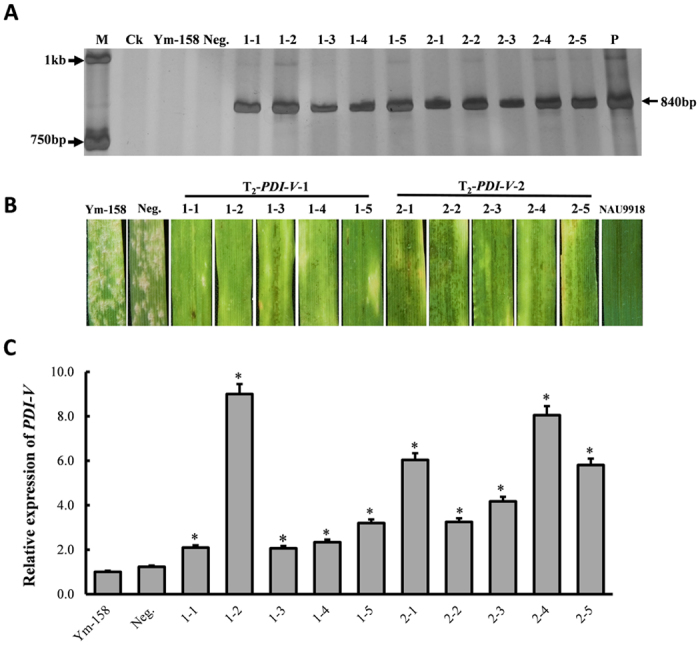
Powdery mildew evaluation and PCR identification of *PDI-V* over-expressing T_2_ plants. (**A**) Detection of positive T_2_ transgenic plants by PCR amplification of *PDI-V* using *pBI220:PDI-V* as positive control. M, DNA marker DL2kb; CK, ddH_2_O; Ym-158, Yangmai 158; Neg., negative T_2_ transgenic plant, P, *pBI220:PDI-V* plasmid. Arrows on the left side of the image indicate the size of the two bands of the 2 kb DNA marker; arrow on the right side shows the size and amplification of the *PDI-V*-specific band. (**B**) Disease responses of T_2_ plants evaluated at the seedling stage following inoculation of detached leaves, using Yangmai 158 and Nannong-9918 (NAU 9918) as susceptible and resistant controls. (**C**) Over-expression of *PDI-V* in T_2_-transgenic plants assayed by qRT-PCR using *Tubulin* as internal control to normalize the quantification data. Significance was determined according to paired sample t-tests. *p < 0.05.

Stable transgenic plants overexpressing *PDI-V* in Yangmai 158 were generated by particle bombardment. Transgenic plants T_0_ to T_2_ generation plants were identified. PCR using a combined primer pair CaMV35S-F & PDI-V-R (Supplementary Table S2) confirmed that 11 regenerated plants were positively transformed. Stable transgenic lines PDI-V-T_2_-1 and PDI-V-T_2_-2, showed enhanced seedling and adult resistance to *Bgt* compared to non-transformed Yangmai 158 (Fig. 5A). At seedling stage both over-expressing lines had infection type (IT) 0, whereas Yangmai 158 was fully susceptible (IT 4). At the adult stage, a few *Bgt* pustules were observed on the *PDI-V* over-expressing lines but far less than on the Yangmai 158 control. In a qRT-PCR assay the expression levels of the *PDI-V* transgene in the two transgenic lines were increased by 2- and 9-fold relative to the non-transgenic control (Fig. 5C).

**Figure 6:**
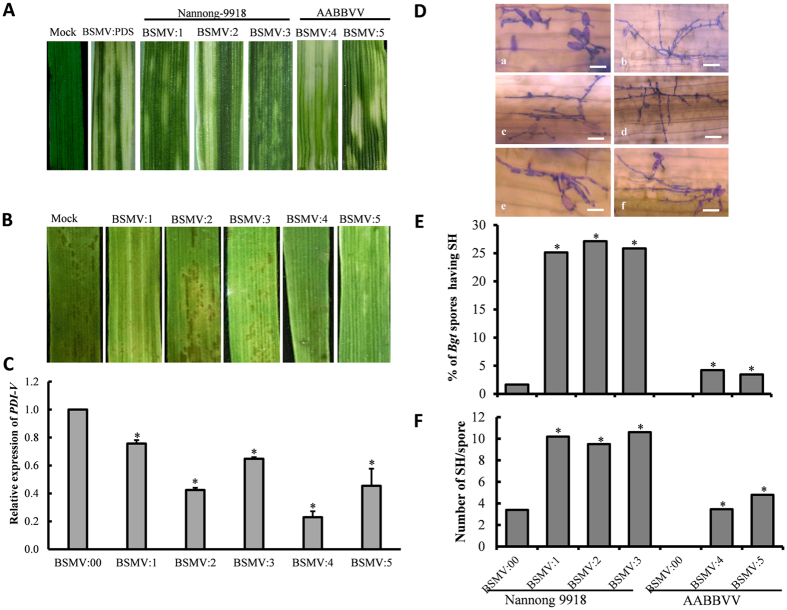
Functional characterization of *PDI-V* in *Bgt*-infected Nannong 9918 and *H. villosa-T. turgidum* amphiploid (AABBVV) by BSMV-mediated VIGS. (**A**) Typical photo bleaching of *BSMV:PDS*-infected was first observed on the third leaf at 9 dpi. Photographs show the fourth leaves at 15 dpi. (**B**) Disease responses at 7 dpi of *Bgt* inoculation on the fourth leaves of *BSMV:PDI-V*-silenced plants. (**C**) Assessment of silencing efficiency of *BSMV:PDI-V* by qRT-PCR assay. The fifth leaves of *PDI-V*-silenced plants were sampled; *BSMV:00* infected plant were used as the control. Data were normalized using the *Tubulin* gene as an internal control. Each point represents the mean of three replicates. Bars indicate SD, *p < 0.05. (**D**) Microscopic observation of fungal growth in *BSMV:00 (a)* and *BSMV:PDI-V* infected plants (b–d from Nannong 9918; e,f from the AABBVV amphiploid). Scale bar = 30 μm (**E**) Comparison of *Bgt* spores producing SH between *BSMV:00*- and *BSMV:PDI*- infected plants (BSMV:1 to BSMV:3, Nannong 9918; BSMV:4 and BSMV:5, AABBVV). (**F**) Numbers of individual fungal spores producing secondary hyphae were counted from whole detached leaves of each genotype and compared with BSMV:00. *P < 0.05 according to Student’s t-test.

VIGS was used to silence *PDI-V* and its wheat orthologs in the *T. durum-H. villosa* amphiploid and wheat cv Nannong 9918. Typical mild chlorotic mosaic symptoms appeared on the 4^th^ leaves of all the BSMV infected plants at 9 dpi, and photobleaching was observed only in plants infected with BSMV:PDS (Fig. 6A). Leaves of plants infected with BSMV:PDI-V sampled at 15 dpi were placed on media containing 6-benzylaminopurine (6-BA) and inoculated with *Bgt*; qRT-PCR showed that *PDI-V* transcripts were significantly decreased in silenced plants compared to the control (Fig. 6C). At 7 dpi no visible powdery mildew symptoms were present on the leaves of both resistant lines (Fig. 6B). However, there were differences in fungal development between the lines with respect to the percentages of *Bgt* conidia producing secondary hyphae (SH) and numbers of SH produced by individual conidia. The percentage of *Bgt* conidia producing SH was significantly higher in *PDI*-silenced leaves of Nannong 9918 than in the amphiploid (Fig. 6E). Similarly, the number of SH produced by individual conidia in *PDI-V*-silenced leaves was also higher (Fig. 6F). These results indicated that *PDI*- silencing reduced the level of resistance in these lines. The haustorial indices were higher in Nannong 9918 than in the *T. durum-H. villosa* amphiploid. We speculate this may due to other genes affecting powdery mildew response in the amphiploid. To obtain a better understanding of the relationships of *PDI-V*, *CMPG1-V* and *Pm 21*, *PDI* expression levels were profiled in plants over-expressing *CMPG1-V* and *Stpk-V* plants (T_2_-generation). The results showed that the expression of *PDI* was greatly induced in both over-expressing lines after *Bgt* inoculation (Supplementary Fig. S4) supporting our hypothesis that decreased resistance in silenced plants was associated with lack of *PDI* expression.

### Enhanced H_2_O_2_ production may contribute to inhibited *Bgt* development

**Figure 7:**
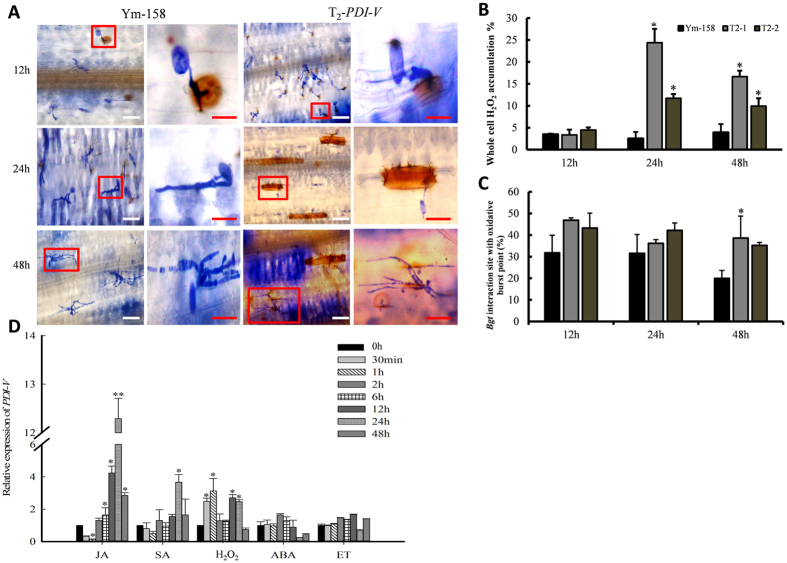
Accumulation of H_2_O_2_ at the *Bgt* infection sites in wheat epidermal cells. (**A**) Microscopic view of H_2_O_2_ production in wheat leaf epidermal cells. Two-week-old seedlings were inoculated with *Bgt* and sampled at different time points as indicated. H_2_O_2_ accumulation was detected by *in situ* histochemical staining using DAB and observed in bright field under an Olympus microscope. Enlarged views of marked red boxes in lanes 1 and 3 (scale bar = 30 μm) are shown in lanes 2 and 4. Scale bar = 20 μm. (**B,C**) Percentage of cells with H_2_O_2_ accumulation throughout the entire cell, and with accumulation only around the infection sites in Yangmai 158 and *PDI-V*-over-expressing plants. (**D**) Expression pattern of *PDI-V* in response to exogenous hormones. Each result is the mean of three independent biological repeats; bars show standard deviations. The *Tubulin* gene was used as an internal control to normalize qRT-PCR values. Asterisks indicate a significant difference (P < 0.05) from the control (0 hpi) based on Student’s t-tests. JA, methyl jasmonate; SA, salicylic acid; H_2_O_2_, hydrogen peroxide; ET, ethylene.

H_2_O_2_ is the key driver of ROS production and signaling that regulates interaction between the SA, JA and ethylene defense pathways in many plants and also in powdery mildew resistance^33^,^34^. H_2_O_2_ treatment triggered immediate expression of *PDI-V*; increased expression was detected at 30 min and 1 h, and significant up-regulation continued at 24 h and 48 h (Fig. 7D). T_2_ *PDI-V* transgenic plants and Yangmai 158 were DAB-stained to measure H_2_O_2_ production and accumulation at different time points within and around *Bgt*-infected cells (Fig. 7A). At 24 and 48 hpi the percentages of H_2_O_2_ accumulation in PDI-V-T_2_-2 were significantly higher than in Yangmai 158. While no significant difference was observed at 12 hpi (Fig. 7B), the percentages of H_2_O_2_ accumulation at 48 hpi were much higher in T_2_-trangenic plants than Yangmai 158 (Fig. 7C). We speculate that overexpression of *PDI-V* induced delayed cell death following pathogen penetration, resulting in improved powdery mildew resistance.

### *PDI-V* expression is up-regulated by different stresses and phytohormone treatments

Expression profiling of *PDI-V* in response to salt (200 mM NaCl), cold (4 °C) and heat shock (40 °C) were assayed by qRT-PCR. All three stresses induced *PDI-V* expression changes (Supplementary Fig. S3). NaCl treatment induced an almost twofold increase in expression of *PDI-V* in *H. villosa* leaves after 3 h. Expression then decreased slightly to a constant level of about 1.75-fold until 12 h, decreased somewhat at 24 h and it was again up-regulated to 1.76-fold at 48 h (Supplementary Fig. S3A). After 24 h exposure of *H. villlosa* at 4 °C, *PDI-V* expression was sharply up-regulated and reached a maximum level of about 3-fold higher than the control (Supplementary Fig. S3C). Heat shock induced a 3-fold up-regulation at 1 h, and maximum 11-fold level at 6 h, before decreasing to the pre-treatment level at 48 h (Supplementary Fig. S3D).

Expression patterns of *PDI-V* in response to treatments by phytohormones (MethJA, ethylene, ABA, SA) were investigated by qRT-PCR. With SA treatment, no significant change was observed until 24 h post treatment when the gene expression reached a maximum level, then decreased and finally back to the pre-treatment level. *PDI-V* in response of MeJA treatment was significantly down-regulated at 30 min, then increased gradually from 2 h and reached a maximum level (12-fold) at 24 h and was maintained at relatively high level of up-regulation for the remainder of the experiment. Ethylene and ABA treatments induced no significant changes in *PDI-V* expression level (Fig. 3D).

**Figure 8:**
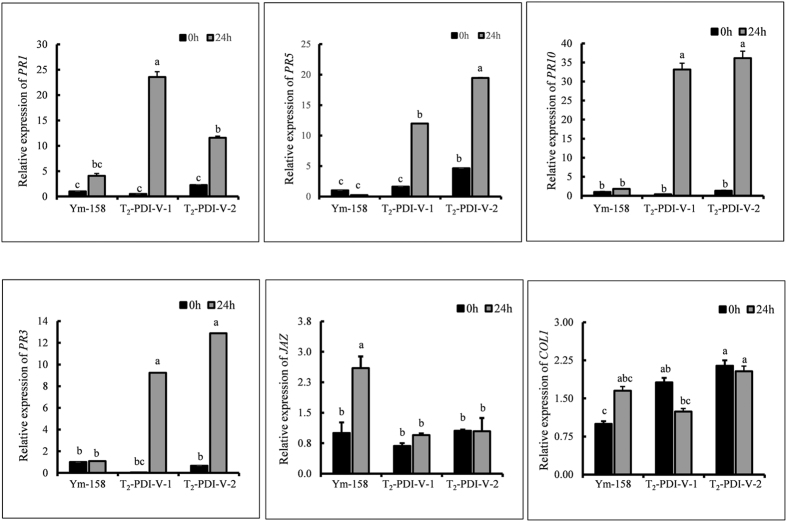
Expression patterns of pathogenesis-related (*PR*) genes in *Bgt*-inoculated leaf tissues of Yangmai 158 and *PDI-V* over-expressing T_2_ lines at 0 and 24 hpi. Each result is the mean of three independent biological repeats; bars shows standard deviations. The *Tubulin* gene was used as an internal control to normalize qRT-PCR values. Lowercase letters represent statistically significant differences at p = 0.05 (one way ANOVA).

Expression of some *PR* genes was studied in over-expressing T_2_ plants to investigate the mechanism of resistance conferred by *PDI-V*. The results showed that expression of *PR1*, *PR5* and *PR10* were significantly up-regulated at 24 hpi relative to the non-transgenic Yangmai 158 control (Fig. 8). Expression of *PR1*, the key marker for SA-systemic acquire resistance (SAR) was also significantly higher in Yangmai 158 after inoculation, however in *PDI-V* transgenic lines it was much higher. The expression pattern of *PR3*, the marker for JA-signaling pathway was also up-regulated in *PDI-V* over-expressing lines, but expressions of *JAZ* and *COL1* were unchanged (Fig. 8). From these results we speculated that *PDI-V* might use SA to produce resistance responses to powdery mildew.

## Discussion

Very little is reported in literature about the participation of *PDI* genes in plant disease response. Some studies suggest a role of cell surface PDIs in molecular transportation in defense-signaling cascades, for example the movement of nitric oxide across cell walls^27^,^28^,^29^. Recent work with barley proved that an *HvPDIL5-1* variant conferred broad spectrum resistance to many Bymoviruses strains^21^. Only one study in wheat reported involvement of a *PDI* gene in defense against the hemibiotrophic fungal pathogen (*M. graminicola*). It found immediate induction of *PDI* in resistant wheat cultivars challenged with the fungus and suggested that PDI may be integral to the repertoire of mechanisms that host plants have evolved to suppress highly destructive, energy-consuming processes accompanying hypersensitive responses^25^. Here we report a functional analysis of *PDI* from the wheat wild relative *H. villosa* in combating wheat powdery mildew.

A Y2H assay and BiFC proved that PDI-V interacted with CMPG1-V, reported previously as a positive regulator of powdery mildew resistance^1^. As an E3 ligase, one possibility was that CMPG-mediated powdery mildew resistance involved triggering of degradation of one or more target proteins^35^. However, we found that PDI-V resides in the ER and is mono-ubiquitinated by CMPG1-V without detectable degradation. Thus PDI-V is an interaction protein of CMPG-V but is not its degradation target. In the host-pathogen interaction systems in animals, PDI not only processes antigens but it is also involved in transferring pathogen antigens from the ER to the cytosol by an ER-associated degradation system for further degradation by proteasomes^22^. We therefore suggest that CMPG1-V might be processed by PDI-V in the ER lumen before being secreted to the cytoplasm as the ER localization of CMPG1-V was already proved^1^.

*PDI-V* is located in chromosome 5VL. Bioinformatic analysis showed high syntenic conservation of the gene location across homologues in monocots (rice, Brachypodium and barley) including the A, B and D sub-genomes of hexaploid wheat^36^,^37^. d’Aloisio *et al.*^19^ characterized the *PDI* genes in bread wheat and grouped them into eight clades. *PDI-V* has the highest similarity with group V (containing two genes designated as *TaPDIL5-1a* and *TaPDIL5-1b*), which were also located on homeologous group 5 wheat chromosomes.

The protein structure of PDI-V is also highly conserved across the monocots in terms of structure, domain number and active thioredoxin sites which are characterized by presence of three domains (*a*, *a′* and *b*), two thioredoxin-active sites and a modified ER retention NDEL signal at the C-terminus. The PDIs having such structural features are known as PDI-a-P5 due to close homology with human P5 PDI. Domain a is present on the N-terminus while a′ is located almost in the middle of PDI-V protein. Studies on the evolution of PDI domains suggested that the C-terminus TRX domain evolved from duplication of the N-terminus TRX domain present in all classes of PDI enzymes^38^.

Single-cell TOA of full-length of *PDI-V* clearly demonstrated that HI, which represents a measure of the compatible interaction between the host and *Bgt*, was reduced significantly in transformed plants compared to Yangmai 158. The multilobe shape of the *Bgt* haustorium not only increases the surface area but also facilitates nutrient absorption from the host cell^39^. Thus a decrease in HI shows that *PDI-V* restricts *Bgt* in establishing contact with the host, ultimately leading to a lower disease level. Our results from single-cell TOA showed that domain a of PDI-V is critical in restraining *Bgt* growth. Over-expression of domain a mutant gene *PDI-V*^*m*^ did not improve resistance compared to over-expression of the wild type gene. This implies that catalytic site in TXR domains present in *PDI-V* has chaperon activity that regulates the physiological function of cells in a complex way.

To gain further insight into *PDI-V* functions in the defense pathway of *Bgt*, we knocked down the gene in two different lines; one having a complete VV genome i.e*. H. villosa-T. turgidum* amphiploid (AABBVV) and wheat cv Nannong 9918 having translocation chromosome 6VS/6AL containing *Pm21*. *Pm21* confers broad spectrum resistance to *Bgt* in China^2^. The purpose of silencing *PDI* in Nannong 9918 was to investigate a possible functional relationship between the two genes. *PDI-V* silencing was insufficient to produce visible disease symptoms on knockdown leaves of both genotypes, but microscopic observations on numbers of germinating *Bgt* conidiospores producing SH per unit area and numbers of SH produced per spore were much higher in Nannong 9918 than in the *H. villosa-T. turgidum* amphiploid. One explanation for this is the presence of additional genes affecting powdery mildew response in the amphiploid than in Nannong 9918. Moreover, high expression of *PDI* in *CMPG1-V* and *Stpk-V* transgenic wheat plants provided preliminary indications that *Pm21* might mediate the PDIs and CMPGs to regulate defense responses. During pathogen infection, ROS production provides the hallmark for successful recognition of pathogen and activation of defense mechanism in plants^40^. The first and quickest response is an oxidative burst to inhibit pathogen attack mainly by virtue of H_2_O_2_ production and consequently act as a signal molecule for the induction of many defensive genes^41^,^42^. Our results confirmed rapid accumulation of H_2_O_2_ at *Bgt* infection sites in transgenic plants as well as in the receptor Yangmai 158. Although initially there was no statistically significant difference in H_2_O_2_ production between *PDI-V* transgenic plants and Yangmai 158 but still the accumulation of H_2_O_2_ in transgenic plants was higher than in Yangmai 158. The whole cell oxidative burst in response to *Bgt* attack was significantly higher in transgenic plants than in Yangmai 158 at 24 hai and thereafter. From these results we supposed that H_2_O_2_ initially functioned as a signal to trigger *PDI-V* induction, which upon activation produced H_2_O_2_ to reduce the *Bgt* growth. A previous study also detected rapid H_2_O_2_ accumulation in wheat mesophyll cells during incompatible *Bgt* interactions, suggesting signaling behavior in defense against *Bgt*^43^. Like the powdery mildew resistance gene *Stpk-V, PDI-V* was also triggered by exogenous H_2_O_2_ application and *Bgt* inoculation^2^. Expression of *PR1*, *PR3*, *PR5* and *PR10* were up-regulated in *PDI-V* transgenic plants, suggesting that PDI-V may use SA signaling to inhibit fungal growth. It has been reported that high expression of *PR1*, *PR2*, *PR5* and *PR10* are consequences of SA-dependent signaling responses in resistance to biotrophic pathogens in many plants species including wheat and *Arabidopsis*^44^,^45^,^46^. Although expression of *PR1*, the key marker for SA-systemic acquired resistance (SAR), has also increased significantly in Yangmai 158 after inoculation but it was much higher in *PDI-V* transgenic lines. This is in accordance with previous findings, as it is a well understood phenomenon that expression of *PR1*, and even *PR5*, is much higher in incompatible than in compatible interaction^47^. In *PDI-V* over-expressing plants, high expression of *PR3* was observed. In many plants, *PR3* has inhibitory role against invading fungal pathogens and can increase the *PR1* expression level, which lead to enhanced disease resistance^45^,^48^.

*PDI-V* was also up-regulated under abiotic stresses salinity, cold and heat shock. It was shown previously that both pathogen infection and abiotic stresses produced unfolded protein stress conditions^49^, which results in triggering of an ER-quality control (ER-QC) mechanism^50^. The ER-QC system comprised an SDF2-ERdj3b-BIP complex, the calreticulin/calnexin cycle, and disulfide isomerase (PDI)^51^. However, the degree to which *PDI-V* can elevate abiotic stress needs further study.

In conclusion, the present study reports for the first time function-based evidence of involvement of *PDIs* in disease resistance in wheat. The catalytic site of thioredoxin domains in *PDI-V* provides chaperon activity in restricting *Bgt* penetration of host cells in a complex regulatory mechanism. PDI-V may function coordinately with Stpk-V and CMPG1-V, and its activity involves the H_2_O_2_ pathway. Future studies will be focused on the functional analysis of the TXR domain and to determine the exact connection between PDI-V and CMPG1-V during host infection.

## Materials and Methods

### Plant materials, growth conditions and chemical treatments

*Haynaldia villosa* (genome VV, accession no. 91C43) was introduced from Cambrige, UK. A *Triticum durum-H. villosa* amphiploid (genome AABBVV, accession no. NAU 201), a set of *T. aestivum*-*H. villosa* addition lines (DA1V to DA7V, accession no. NAU307 to NAU313, each containing one pair of chromosomes from *H. villosa* in Chinese Spring wheat, and two *T. aestivum-H. villosa* whole arm translocation lines T5VS/5DL and T5VL/W (CINAU-61 and CINAU-158) were developed by the Cytogenetics Institute, Nanjing Agricultural University (CINAU). Wheat cv. Yangmai 158 (moderately susceptible to *Bgt*) and Nannong 9918 (containing *Pm21*, and immune to *Bgt*) were used as receptors for transient and/or stable transformation and virus-induced gene silencing (VIGS). The conditions for plant growth and *Bgt* inoculations were as described in Xing *et al.*^52^.

To study the expression level of *PDI-V* in response to different treatments, *H. villosa* seedlings at the two-leaf stages were either inoculated with *Bgt*, or moved to 4 °C or 40 °C conditions for cold and heat shock stress treatments, or dipped into 200 mM NaCl for salinity stress. Leaf tissue samples were collected at 0 h, 30 min, 45 min, 1 h, 2 h, 6 h, 12 h, 24 h, 48 h and 72 h post treatments. Parallel mock-inoculated controls were treated with sterile water. Treatments with exogenous hormones or signal molecules were performed as described in Xing *et al.*^52^. Three biological repeats were used for each assay. All samples were rapidly frozen in liquid nitrogen and stored in an ultra-freezer (−80 °C) until use.

### Total RNA extraction and quantitative reverse transcription-PCR (qRT-PCR)

Total RNA was extracted using Trizol (Invitrogen, Carlsbad, CA) following the instructions of the manufacturer. Primer oligo (dT)_18_ was used to reversely transcribe 2 μg of total RNA into cDNA using AMV reverse transcriptase (Takara) in RT-PCR. The expression pattern of *PDI-V* was analyzed using a SYBR Green/Fluorescent qPCR master mix (Takara) on a Roche-480 system (Roche), and the wheat *Tubulin* gene was used as the internal control. The qRT-PCR program started with denaturation at 95 °C for 1 min, followed by 40 amplification cycles programmed as 95 °C 5 sec, 57 °C 30 sec, and 72 °C 30 sec. The CT values for target and standard control genes were retrieved and the comparative threshold 2^−∆∆CT^ method was applied to quantify the relative expression of given genes^53^. All reactions were conducted in three biological replicates with three technical repeats for each replication. Double distilled H_2_O was used as template for negative controls. All primers used in the study are listed in Supplementary Table S2.

### Yeast two-hybrid protein-protein interaction

A yeast two-hybrid (Y2H) *H. villosa* cDNA library was constructed by Li^32^. To investigate protein to protein interaction between CMPG1-V and PDI-V, GAL-4-based Y2H was performed in the HF7c strain of *S. cerevisiae* that harbored reporter genes lacZ and HIS3. The complete ORF of CMPG1-V was fused with the GAL4 DNA binding domain (DNA-BD) of protein expression vector pGBKT7 as a bait while the PDI-V cDNA sequence was inserted as prey into a pGADT7 vector harboring an activation domain (AD) using the *Sma*I and *Bam*HI cloning sites. The resulting constructs and empty vectors were transformed into yeast strain *HF7c* as described by Xie *et al.*^54^. The transformation mixtures were plated on yeast drop-down selection media (SD-LTH) deprived of leucine (Leu), tryptophan (Trp) and histidine (His) but supplemented with 10 mM 3-amino-1,2,4,triazole (3-AT) to reduce the appearance of false positive colonies. In addition the same combination of transformation mixtures were plated on SD-Leu-Trp medium (SD-LT) as a control to check for normal growth of the yeast colonies. Proteins interactions were evaluated on the basis of growth status of yeast cells on SD-LTH media after incubation at 30 °C for 3 days.

### Bimolecular fluorescence complementation (BiFC) assay

A BiFC assay was performed as described by Walter *et al.*^55^. In short, coding regions of *CMPG1-V* and *PDI-V* were amplified by PCR using specific primers (Supplementary Table S1) and cloned into pSPYNE-35S and pSPYCE-35S, respectively. The resulting constructs (CMPG1-V-nYFP and PDI-V-cYFP) were transformed into onion epidermal cells with a BIOLISTIC-PDS-1000/He particle delivery system (Bio-Rad) at a pressure of 1100 p.s.i. The transformed onion epidermal layers were incubated in darkness at 22 °C for 16 h before detection of yellow florescence signals by confocal microscopy (Leica Microsystem SP2).

### *In vitro* pull-down assay

To purify GST-PDI-V protein, the full-length ORF of PDI-V was cloned into a pGEX4 vector using the restriction sites *Bam*HI and *Sma*I. Resultant GST-tagged PDI-V protein was expressed in the *Escherichia coli* strain BL21 (DE3) and purified by affinity chromatography using glutathione sepharose beads. Generation of the MBP-CMPG1-V construct and protein purification were performed as described by Zhu *et al.*^1^. For the pull-down assay about 10 μg of the MBP-PDI-V and MBP alone (control) proteins were incubated with 10 μL of pre-washed amylose beads into different microfuge tubes containing 1 mL of pull down buffer (20 mM TRIS-HCl, pH 7.5, 1 mM β-mercaptoethanol, 3 mM EDTA, 150 mM NaCl, and 1% NP-40) for 1 h at 4 °C with gentle shaking. Incubated beads were centrifuged at 2000 rpm for 1 min before second incubation with 10 μg of GST-PDI-V protein in 1 mL of pull down buffer for 30 min at room temperature with constant gentle shaking. After incubation the beads were harvested, washed once with PBS buffer containing 500 mM NaCl and subsequently washed five times with the same buffer containing 135 mM of NaCl. The bound protein complex retained on the beads was extracted by boiling the beads in 10 μL of 2 × SDS-PAGE loading buffer and finally analyzed by western blotting using α-GST antibody.

### Chromosome location of *PDI-V*

To determine the chromosome location of *PDI-V*, *H. villosa*, *T. turgidum-H. villosa* amphiploid, a set of all seven addition lines (DA1V to DA7V), and Chinese Spring were used for PCR using primer pair PDI-V-40F/R, designed from the *PDI* homolog from the sequenced D-genome of *Ae. tauschii* (Supplementary Table S2). PCR were performed in 25 μL reaction volumes including 1 × PCR buffer, 2 mmol/l MgCl_2_, 0.15 mmol/l dNTPs, 20 ng of each primer, 2 μL template and 1U r-*Taq* DNA polymerase (Takara, Japan). Conditions for thermal cycling were: denaturing at 94 °C for 5 min, followed by 94 °C 45 s, 59 °C 45 s, 72 °C for 1 min for 35 cycles, and final extension at 72 °C for 10 min. The amplified products were visualized in 1% agrose gels containing ethidum bromide.

### Sub-cellular localization of *PDI-V*

The coding sequence of PDI-V without a stop codon was amplified with specific primers (PDI-V-580F & R) and cloned into the *Spe*I and *BamH*I sites in front of green fluorescent protein (GFP) under control of the *Cauliflower mosaic virus* (CaMV) 35S promoter in transient expression vector -pAN580. The resultant recombinant pAN580-PDI-V-GFP vector was confirmed by DNA sequencing. For onion bombardment, gold particles (1 mm in diameter) were coated separately with 5 mg of plasmid DNA with the empty vector pAN580 and pAN580-PDI-V-GFP and bombarded with a BIOLISTIC-PDS-1000/He particle delivery system (Bio-Rad) at a pressure of 1100 p.s.i. Onion epidermal peels were placed on solidified half-strength Murashige and Skoog (MS) medium and kept at 22 °C in darkness for 24 h before imaging with a Zeiss LSM 730 microscope.

### *In vitro* ubiquitination and cell-free degradation assays

The purified proteins (MBP-CMPG1-V and GST-PDI-V) were used for *in vitro* ubiquitination assay according to Xie *et al.*^56^ with minor modifications. A final volume of 30 μL was used for each reaction, containing 40 mM Tris-HCl pH 7.4, 5 mM MgCl_2_, 50 mM KCl, 2 mM ATP, 1 mM DTT, 10% glycerol, 200 ng E1, 200 ng E2, 200 ng MBP-CMPG1-V and 600 ng of GST-PDI-V. Reactions were incubated at 30 °C for 1.5 h. Samples were separated on 10% SDS-PAGE gels and ubiquitination was detected by western blotting using anti-GST antibody (Roche).

Cell-free degradation assays were carried out according to García-Cano *et al.*^57^. Briefly, total protein was extracted by grinding wheat leaves in 1 × PBS protein extraction buffer. Sequential centrifugations at 12,000 g for 5 min yielded clear supernatant. Equal concentrations of GST-PDI-V and MBP-CMPG1-V were added to 200 μL of total protein extract supplemented with, or without, the 26S proteasome inhibitor MG132 (40 μM). Samples were taken from both treatments at time intervals of 0, 5, 15 and 30 min and analyzed by western blotting using anti-GST antibody (Roche).

### VIGS assay

To silence *PDI-V*, barley stripe mosaic virus (BSMV)-based infectious vectors were constructed as documented by Wang *et al.*^58^. The partial sequence of *PDI-V* (200 bp) was cloned by specific primer pair PDIV-VIGS-F/R (Supplementary Table S2) and inserted in RNAγ of BSMV. About 2.5 μL of each BSMV RNA *i.e.*, α, β and genetically modified γ mixed with 42.5 μL of 2 × GKP buffer, was inoculated to two-leaf seedlings by gently rubbing the leaves with gloved fingers. BSMV:PDS and BSMV:00 were used as controls. Each assay consisted of 20 seedlings and repeated at least three times. Detached fourth leaves from 10 plants in each treatment were placed on 6-BA media and inoculated with high densities of freshly collected *Bgt* conidiospores. The phenotypes of inoculated leaves were observed and photographed at 10 dpi. To determine the silencing efficiency RNA extracted from fourth leaves of remaining plants was used to measure *PDI-V* expression levels by qRT-PCR.

### Single-cell transient overexpression assay (TOA)

Single-cell TOA is a highly reliable, quick and powerful approach to study defense functions of foreign genes against *Bgt* due to the ectoparasitic nature of pathogen and synchronous restriction of the fungus to penetrated cell only^59^. The TOA of *PDI-V* was performed using a Bio-Rad He/1000 particle delivery system to wheat leaves as described by Shirasu *et al.*^60^. Equal volumes (1 μg/μL) of plasmid DNA (*pBI220:PDI-V*) and pWMB002 vector (containing the *GUS* gene) were mixed before coating tungsten particles. Mixed tungsten particles and plasmids were prepared for 15 shots. Second leaves of one-week-old Yangmai 158 seedlings were firmly placed on the plated media for delivery of mixed plasmids into host cells by gene gun. Delivery of pWMB002 alone was used as control. Bombarded leaves were incubated at 25 °C for 4 to 6 h in darkness, then infected with fresh *Bgt* inoculum and placed in a growth chamber with a 14 h light/10 h darkness photoperiod for 40 h^61^. Leaves were stained for determination of GUS activity, bleached with 90% ethanol, and observed under a microscope to record the haustorial index (HI, percentage of GUS-staining cells with haustoria among all GUS-stained cells invaded by *Bgt*). The assay was repeated twice and significant differences between treatments were analyzed by paired sample t-tests using SPSS software.

### Generation and characterization of *PDI-V* transgenic wheat plants

A point mutation *PDI-V*^*m*^ was created in the catalytic motif of domain *a*, using the Mut Express II Fast Mutagenesis Kit (Vazyme, Nanjing) according to the manufacturer’s recommendations. The coding regions of *PDI-V* and *PDI-V*^*m*^ were amplified by PCR and cloned into the over- expression vector pBI220-HA between the *Bam*HI and *Stu*I cloning sites. The resulting constructs pBI220-HA-PDI-V and pBI220-HA-PDI-V^m^ were co-transformed along with pAHC20 (having a herbicide tolerance marker gene) into immature-embryo derived calli of wheat cv. Yangmai 158 by particle bombardment. Transformed calli were cultured and regenerated into fully developed plants as prescribed by Xing *et al.*^16^. Total genomic DNA of T_0_ transgenic and subsequent generation (T_1_ and T_2_) plants were used to detect the positive plants by amplification with a combined specific primer flanking the CaMV35S promoter and PDI-V, CaMV35S-F and PDI-V-R (Supplementary Table S2).

For characterizing the function of *PDI-V*, detached young leaves from all plants in each generation (T_0_, T_1_ and T_2_) were inoculated with *Bgt* to check the infection types according to Sheng *et al.*^62^. Two T_2_ transgenic lines derived from positive T_0_ plants showing high levels of powdery mildew resistance were selected and grown in the field and greenhouse. Five rows of each T_2_ transgenic line were planted along with the negative control Yangmai 158 (receptor variety). Two rows of highly susceptible cv. Sumai 3 were planted every six rows for generation of *Bgt* inoculum. Disease responses of adult T_2_ plants was recorded using a 0–9 disease scale^64^.

### Detection of ROS

Accumulation of H_2_O_2_ in plant cells was detected by 3, 3′-diaminobenzidine (DAB) staining as described by Xing *et al.*^52^. Leaves of two T_2_-PDI-V transgenic lines and Yangmai 158 were soaked in DAB solution (Bio Basic Inc., Shanghai) for 8 h for uptake and then incubated at 25 °C for an additional 8 h. At 12, 24 and 48 hpi, 1.5 cm leaf segments from each host line were sampled, and boiled in 95% ethanol for 5 min. The cleared leaf segments were mounted in 50% glyercerol and observed under a microscope (Olympus, Japan). Randomly selected 5 leaf segments from each line and at least 100 infection sites on each leaf were examined. Standard deviations were determined and pair sample t-tests were used for statistical analysis.

### *In silico* analysis

The BLAST tool (http://www.ncbi.nlm.nih.gov/blast/) was used to analyze conserved motifs and domain structure of *PDI-V*. The full ORF was predicted by online ORF finder software (http://www.ncbi.nlm.nih.gov/gorf/gorf.html). Retrieved sequences were aligned by the Clustal X 1.83 program using the Gonnet series as protein weight matrix along with other parameters set to a 30% divergent sequence delay and 10 gap open penalty. The phylogenetic tree was constructed using the neighbour-joining method in MEGA 4.0 software. For this all the gaps in the alignment were deleted prior to estimation of distance matrices calculated according to a Poisson correction amino acid model. Statistical significance of phylogenetic tree was tested with 1,000 bootstrap replicates.

## Additional Information

**How to cite this article**: Faheem, M. *et al.* A disulphide isomerase gene (*PDI-V*) from *Haynaldia villosa* contributes to powdery mildew resistance in common wheat. *Sci. Rep.* **6**, 24227; doi: 10.1038/srep24227 (2016).

## Supplementary Material

Supplementary Information

## References

[b1] Zhu Y. *et al.* E3 ubiquitin ligase gene *CMPG1–V* from *Haynaldia villosa* L. contributes to powdery mildew resistance in common wheat (*Triticum aestivum* L.). Plant J. 84, 154–168 (2015). 26287740 10.1111/tpj.12966

[b2] Cao A. *et al.* Serine/threonine kinase gene *Stpk-V*, a key member of powdery mildew resistance gene *Pm21*, confers powdery mildew resistance in wheat. Proc. Natl. Acad. Sci. 108, 7727–7732 (2011). 21508323 10.1073/pnas.1016981108 PMC3093467

[b3] Dodds P. N. & Rathjen J. P. Plant immunity: towards an integrated view of plant–pathogen interactions. Nat. Rev. Genet. 11, 539–548 (2010). 20585331 10.1038/nrg2812

[b4] Beck M., Heard W., Mbengue M. & Robatzek S. The INs and OUTs of pattern recognition receptors at the cell surface. Curr. Opin. Plant Biol. 15, 367–374 (2012). 22664220 10.1016/j.pbi.2012.05.004

[b5] Bozkurt T. O., Schornack S., Banfield M. J. & Kamoun S. Oomycetes, effectors, and all that jazz. Curr. Opin. Plant Biol. 15, 483–492 (2012). 22483402 10.1016/j.pbi.2012.03.008

[b6] Rafiqi M., Ellis J. G., Ludowici V. A., Hardham A. R. & Dodds P. N. Challenges and progress towards understanding the role of effectors in plant–fungal interactions. Curr. Opin. Plant Biol. 15, 477–482 (2012). 22658704 10.1016/j.pbi.2012.05.003

[b7] Chen P., Qi L., Zhou B., Zhang S. & Liu D. Development and molecular cytogenetic analysis of wheat-*Haynaldia villosa* 6VS/6AL translocation lines specifying resistance to powdery mildew. Theor. Appl. Genet. 91, 1125–1128 (1995). 24170007 10.1007/BF00223930

[b8] Chen L. & Hellmann H. Plant E3 ligases: flexible enzymes in a sessile world. Mol. Plant 6, 1388–1404 (2013). 23307436 10.1093/mp/sst005

[b9] Liu J. *et al.* The U-box E3 ligase *SPL11/PUB13* is a convergence point of defense and flowering signaling in plants. Plant Physiol. 160, 28–37 (2012). 22659522 10.1104/pp.112.199430 PMC3440206

[b10] Furniss J. J. & Spoel S. H. Cullin-RING ubiquitin ligases in salicylic acid-mediated plant immune signaling. Front. Plant Sci. 6, 154; 10.3389/fpls.2015.00154 (2015). 25821454 PMC4358073

[b11] Freedman R. B., Hirst T. R. & Tuite M. F. Protein disulphide isomerase: building bridges in protein folding. Trends Biochem. Sci. 19, 331–336 (1994). 7940678 10.1016/0968-0004(94)90072-8

[b12] Hatahet F. & Ruddock L. W. Protein disulfide isomerase: a critical evaluation of its function in disulfide bond formation. Antioxid. Redox signal. 11, 2807–2850 (2009). 19476414 10.1089/ars.2009.2466

[b13] Ciaffi M., Paolacci A., Dominici L., Tanzarella O. & Porceddu E. Molecular characterization of gene sequences coding for protein disulfide isomerase (PDI) in durum wheat (*Triticum turgidum ssp. durum*). Gene 265, 147–156 (2001). 11255017 10.1016/s0378-1119(01)00348-1

[b14] Dhanapal A. P. & Porceddu E. Funtional and evolutionary genomics of protein disulphide isomerase (PDI) gene family in wheat and its wild relatives: A review. Annu. Rev. Res. Biol. 3, 935–958 (2013).

[b15] Ondzighi C. A., Christopher D. A., Cho E. J., Chang S.-C. & Staehelin L. A. *Arabidopsis* protein disulfide isomerase-5 inhibits cysteine proteases during trafficking to vacuoles before programmed cell death of the endothelium in developing seeds. Plant Cell Online 20, 2205–2220 (2008). 10.1105/tpc.108.058339 PMC2553623 18676877

[b16] Wang H., Boavida L. C., Ron M. & McCormick S. Truncation of a protein disulfide isomerase, PDIL2-1, delays embryo sac maturation and disrupts pollen tube guidance in *Arabidopsis thaliana*. Plant Cell 20, 3300–3311 (2008). 19050167 10.1105/tpc.108.062919 PMC2630445

[b17] Shimoni Y., Zhu X., Levanony H., Segal G. & Galili G. Purification, characterization, and intracellular localization of glycosylated protein disulfide isomerase from wheat grains. Plant Physiol. 108, 327–335 (1995). 7784507 10.1104/pp.108.1.327 PMC157338

[b18] Ciaffi M., Dominici L., Tanzarella O. & Porceddu E. Chromosomal assignment of gene sequences coding for protein disulphide isomerase (PDI) in wheat. Theor. Appl. Genet. 98, 405–410 (1999).

[b19] d’Aloisio E. *et al.* The protein disulfide isomerase gene family in bread wheat (*T. aestivum* L.). BMC Plant Biol. 10, 101; 10.1186/1471-2229-10-101 (2010). 20525253 PMC3017771

[b20] Kimura S., Higashino Y., Kitao Y., Masuda T. & Urade R. Expression and characterization of protein disulfide isomerase family proteins in bread wheat. BMC Plant Biol. 15, 73; 10.1186/s12870-015-0460-2 (2015). 25849633 PMC4355359

[b21] Yang P. *et al.* *PROTEIN DISULFIDE ISOMERASE LIKE 5-1* is a susceptibility factor to plant viruses. Proc. Natl. Acad. Sci. 111, 2104–2109 (2014). 24481254 10.1073/pnas.1320362111 PMC3926060

[b22] Stolf B. S. *et al.* Protein disulfide isomerase and host-pathogen interaction. Sci. World J. 11, 1749–1761 (2011). 10.1100/2011/289182 PMC3201685 22125433

[b23] Forrester M. T., Benhar M. & Stamler J. S. Nitrosative stress in the ER: a new role for S-nitrosylation in neurodegenerative diseases. ACS Chem. Biol. 1, 355–358 (2006). 17163772 10.1021/cb600244c

[b24] Halloran M., Parakh S. & Atkin J. The role of s-nitrosylation and s-glutathionylation of protein disulphide isomerase in protein misfolding and neurodegeneration. Int. J. Cell Biol. 2013, 10.1155/2013/797914 (2013). PMC3852308 24348565

[b25] Ray S., Anderson J. M., Urmeev F. I. & Goodwin S. B. Rapid induction of a protein disulfide isomerase and defense-related genes in wheat in response to the hemibiotrophic fungal pathogen *Mycosphaerella graminicola*. Plant Mol. Biol. 53, 741–754 (2003). 10.1023/B:PLAN.0000019120.74610.52 15010608

[b26] Giraldo M. C. & Valent B. Filamentous plant pathogen effectors in action. Nat. Rev. Microbiol. 11, 800–814 (2013). 24129511 10.1038/nrmicro3119

[b27] Durner J., Wendehenne D. & Klessig D. F. Defense gene induction in tobacco by nitric oxide, cyclic GMP, and cyclic ADP-ribose. Proc. Natl. Acad. Sci. 95, 10328–10333 (1998). 9707647 10.1073/pnas.95.17.10328 PMC21508

[b28] Delledonne M., Xia Y., Dixon R. A. & Lamb C. Nitric oxide functions as a signal in plant disease resistance. Nature 394, 585–588 (1998). 9707120 10.1038/29087

[b29] Zai A., Rudd M. A., Scribner A. W. & Loscalzo J. Cell-surface protein disulfide isomerase catalyzes transnitrosation and regulates intracellular transfer of nitric oxide. J. Clin. Invest. 103, 393–399 (1999). 9927500 10.1172/JCI4890 PMC407899

[b30] Nekrasov V. *et al.* Control of the pattern‐recognition receptor EFR by an ER protein complex in plant immunity. EMBO J. 28, 3428–3438 (2009). 19763086 10.1038/emboj.2009.262 PMC2776097

[b31] Saijo Y. *et al.* Receptor quality control in the endoplasmic reticulum for plant innate immunity. EMBO J. 28, 3439–3449 (2009). 19763087 10.1038/emboj.2009.263 PMC2776098

[b32] Li Y. Identification of a *CMPG1-V* interaction protein *HvHIPP1* from *Haynaldia villosa* L. and its functional analysis in powdery mildew resistance. *Ph.D Thesis*, Nanjing Agricultural Univeristy (2014).

[b33] Overmyer K., Brosché M. & Kangasjärvi J. Reactive oxygen species and hormonal control of cell death. Trends Plant Sci. 8, 335–342 (2003). 12878018 10.1016/S1360-1385(03)00135-3

[b34] Bouchez O., Huard C., Lorrain S., Roby D. & Balagué C. Ethylene is one of the key elements for cell death and defense response control in the *Arabidopsis* lesion mimic mutant *vad1*. Plant Physiol. 145, 465–477 (2007). 17720753 10.1104/pp.107.106302 PMC2048732

[b35] Gilroy E. M. *et al.* *CMPG1*‐dependent cell death follows perception of diverse pathogen elicitors at the host plasma membrane and is suppressed by *Phytophthora infestans* RXLR effector *AVR3a*. New Phytol. 190, 653–666 (2011). 21348873 10.1111/j.1469-8137.2011.03643.x

[b36] Wu H., Dorse S. & Bhave M. *In silico* identification and analysis of the protein disulphide isomerases in wheat and rice. Biologia 67, 48–60 (2012).

[b37] Zhu C. *et al.* Molecular characterization and expression profiling of the protein disulfide isomerase gene family in *Brachypodium distachyon* L. PLos ONE 9, e94704; 10.1371/journal.pone.0094704 (2014). 24747843 PMC3991636

[b38] Kanai S., Toh H., Hayano T. & Kikuchi M. Molecular evolution of the domain structures of protein disulfide isomerases. J. Mol. Evol. 47, 200–210 (1998). 9694669 10.1007/pl00006377

[b39] Braun U. *et al.* The taxonomy of the powdery mildew fungi. The powdery mildews: a comprehensive treatise, (edsm Belanger R. R. *et al.*) 13–55 (APS press, 2002).

[b40] Li A. *et al.* Transcriptome analysis of H_2_O_2_-treated wheat seedlings reveals a H_2_O_2_-responsive fatty acid desaturase gene participating in powdery mildew resistance. PLos ONE 6, e28810; 10.1371/journal.pone.0028810 (2011). 22174904 PMC3236209

[b41] Torres M. A., Jones J. D. & Dangl J. L. Reactive oxygen species signaling in response to pathogens. Plant Physiol. 141, 373–378 (2006). 16760490 10.1104/pp.106.079467 PMC1475467

[b42] Lenucci M. S., Piro G. & Dalessandro G. In muro feruloylation and oxidative coupling in monocots: a possible role in plant defense against pathogen attacks. Plant Signal. Behav. 4, 228–230 (2009). 19721758 10.4161/psb.4.3.7883 PMC2652537

[b43] Li A. *et al.* Comparative analysis of early H_2_O_2_ accumulation in compatible and incompatible wheat–powdery mildew interactions. Plant Pathol. 54, 308–316 (2005).

[b44] Niu J. S., Liu R. & Zheng L. Expression analysis of wheat *PR-1, PR-2, PR-5* activated by *Bgt* and SA, and powdery mildew resistance. Wheat Crop 27, 1132–1137 (2007).

[b45] Hamamouch N., Li C., Seo P. J., Park C. M. & Davis E. L. Expression of *Arabidopsis* pathogenesis‐related genes during nematode infection. Mol. Plant Pathol. 12, 355–364 (2011). 21453430 10.1111/j.1364-3703.2010.00675.x PMC6640486

[b46] Xin M. *et al.* Transcriptome comparison of susceptible and resistant wheat in response to powdery mildew infection. Genomics Proteomics Bioinformatics 10, 94–106 (2012). 22768983 10.1016/j.gpb.2012.05.002 PMC5054165

[b47] Pritsch C., Muehlbauer G. J., Bushnell W. R., Somers D. A. & Vance C. P. Fungal development and induction of defense response genes during early infection of wheat spikes by *Fusarium graminearum*. Mol. Plant Microbe Interact. 13, 159–169 (2000). 10659706 10.1094/MPMI.2000.13.2.159

[b48] Fiocchetti F. *et al.* Over-expression of a pathogenesis-related protein gene in transgenic tomato alters the transcription patterns of other defence genes. J. Hortic. Sci. Biotechnol. 81, 27–32 (2006).

[b49] Urade R. The endoplasmic reticulum stress signaling pathways in plants. Biofactors 35, 326–331 (2009). 19415737 10.1002/biof.45

[b50] Malhotra J. D. & Kaufman R. J. The endoplasmic reticulum and the unfolded protein respnse. In Seminars in Cell & Developmental Biology. 716–731 (Elsevier, 2007). 18023214 10.1016/j.semcdb.2007.09.003 PMC2706143

[b51] Anelli T. & Sitia R. Protein quality control in the early secretory pathway. EMBO J. 27, 315–327 (2008). 18216874 10.1038/sj.emboj.7601974 PMC2234347

[b52] Xing L. *et al.* The *Hv-SGT1* gene from *Haynaldia villosa* contributes to resistances towards both biotrophic and hemi-biotrophic pathogens in common wheat (*Triticum aestivum* L.). PLos ONE 8, e72571; 10.1371/journal.pone.0072571 (2013). 24019872 PMC3760960

[b53] Livak K. J. & Schmittgen T. D. Analysis of relative gene expression data using real-time quantitative PCR and the 2^−ΔΔCT^ method. Methods 25, 402–408 (2001). 11846609 10.1006/meth.2001.1262

[b54] Xie Q., Sanz-Burgos A. P., Guo H., García J. A. & Gutiérrez C. GRAB proteins, novel members of the NAC domain family, isolated by their interaction with a geminivirus protein. Plant Mol. Biol. 39, 647–656 (1999). 10350080 10.1023/a:1006138221874

[b55] Walter M. *et al.* Visualization of protein interactions in living plant cells using bimolecular fluorescence complementation. Plant J. 40, 428–438 (2004). 15469500 10.1111/j.1365-313X.2004.02219.x

[b56] Xie Q. *et al.* SINAT5 promotes ubiquitin-related degradation of NAC1 to attenuate auxin signals. Nature 419, 167–170 (2002). 12226665 10.1038/nature00998

[b57] García-Cano E., Zaltsman A. & Citovsky V. Assaying proteasomal degradation in a cell-free system in plants. J. Vis. Exp. 85, 10.3791/51293 (2014). PMC4090386 24747194

[b58] Wang X. *et al.* Establishment of an effective virus induced gene silencing system with *BSMV* in *Haynaldia villosa*. Mol. Biol. Rep. 37, 967–972 (2010). 19714483 10.1007/s11033-009-9766-1

[b59] Schweizer P., Pokorny J., Abderhalden O. & Dudler R. A transient assay system for the functional assessment of defense-related genes in wheat. Mol. Plant Microbe Interact. 12, 647–654 (1999).

[b60] Shirasu K., Nielsen K., Piffanelli P., Oliver R. & Schulze‐Lefert P. Cell‐autonomous complementation of *mlo* resistance using a biolistic transient expression system. Plant J. 17, 293–299 (1999).

[b61] Ai-li L. A transient expression system for the functional assessment of early response genes on the powdery mildew infected barley or wheat leaves. Agric. Sci. China 2, 1061–1068 (2003).

[b62] Xing L. *et al.* Transformation of wheat *thaumatin-like protein* gene and diseases resistance analysis of transgenic plants. Acta Agron. Sin. 34, 349–354 (2008).

[b63] Sheng B. Grades of resistance to powdery mildew classified by different phenotypes of response in the seeding stage of wheat. Plant Prot. 1, 49 (1988).

[b64] Jakobson I., Peusha H., Timofejeva L. & Järve K. Adult plant and seedling resistance to powdery mildew in a *Triticum aestivum* × *Triticum militinae* hybrid line. Theor. Appl. Genet. 112, 760–769 (2006). 16362813 10.1007/s00122-005-0181-2

