# Supplementary material for: A disulphide isomerase gene (PDI-V) from Haynaldia villosa contributes to powdery mildew resistance in common wheat
Source: Sci Rep. 2016 Apr 13;6:24227. doi: 10.1038/srep24227 (PMC4829865; doi:10.1038/srep24227)
Supplement: Supplementary Information [file srep24227-s1.pdf]

A disulphide isomerase gene (*PDI-V*) from *Haynaldia villosa* contributes to powdery mildew resistance in common wheat

Muhammad Faheem[1,+], Yingbo Li[1,2+], Muhammad Arshad[1], Cheng Jiangyue[1], Zhao Jia[1], Zongkuan Wang[1], Jin Xiao[1], Haiyan Wang[1], Aizhong Cao[1], Liping Xing[1], Feifei Yu[3], Ruiqi Zhang[1], Qi Xie[3], Xiue Wang[1,*]

* Corresponding author: Xiue Wang

Email Address: xiuew@njau.edu.cn

Affiliation: [1]The State Key Laboratory of Crop Genetics and Germplasm Enhancement, Cytogenetics Institute, Nanjing Agricultural University/JCIC-MCP, Nanjing, Jiangsu 210095, P.R. China

[2] Biotech Research Institute, Shanghai Academy of Agricultural Sciences, Shanghai Key Laboratory of Agricultural Genetics and Breeding, Shanghai 201106, China

[3] Institute of Genetics and Developmental Biology, Chinese Academy of Sciences, Beijing 100101, China

[+] These authors contributed equally to this work.

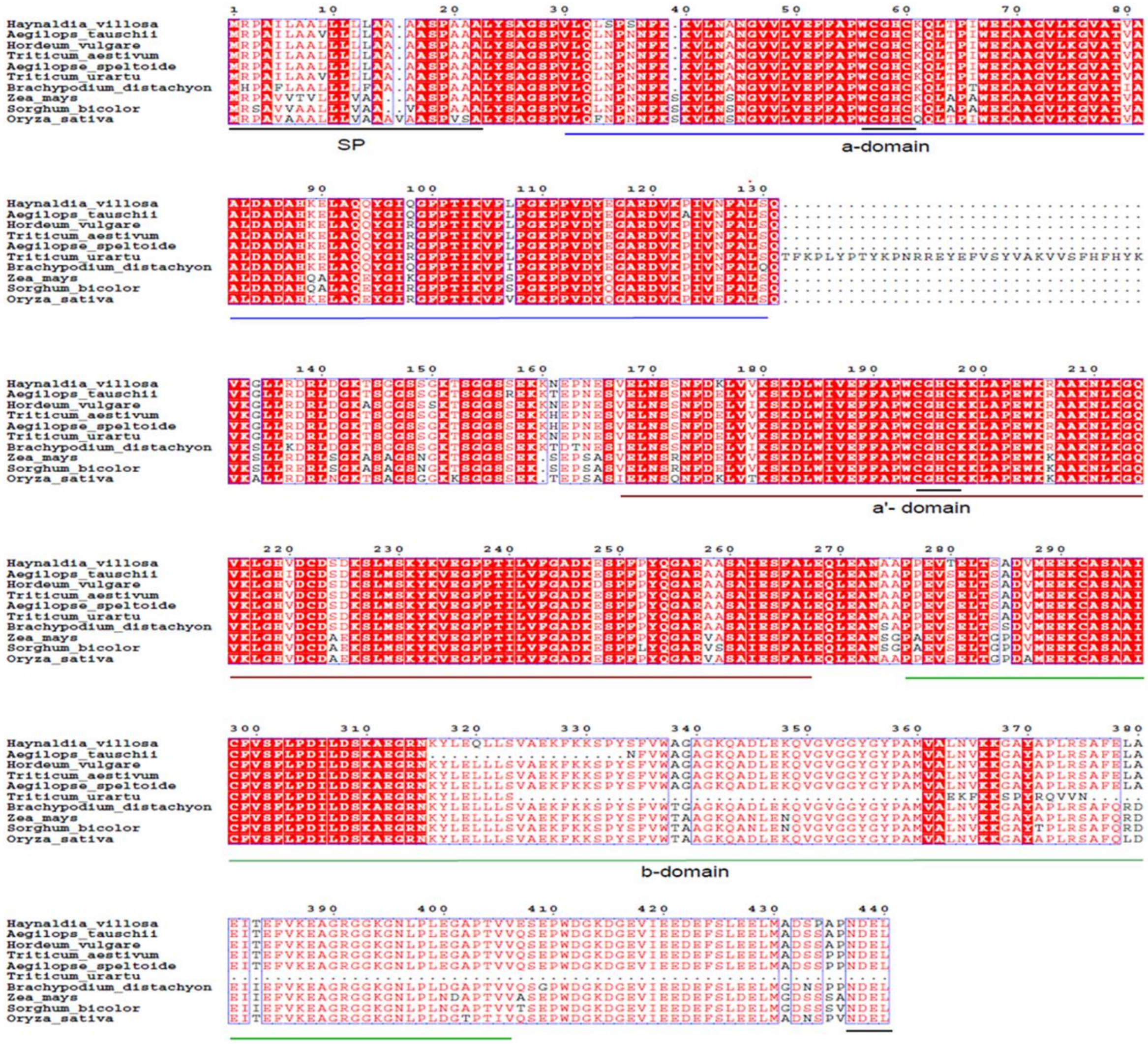

Supplementary Figure S1: Multiple alignment of PDI-V amino acids. Identical and similar amino acids residues are shaded in red and white background respectively. Domains a, a′ and b are underlined with blue, brown and green colors respectively while signal peptide (SP) motif at N-terminus, conserved CGCH motifs in a and a′ domains and NDEL motif at C-terminus are underlined with black bars.

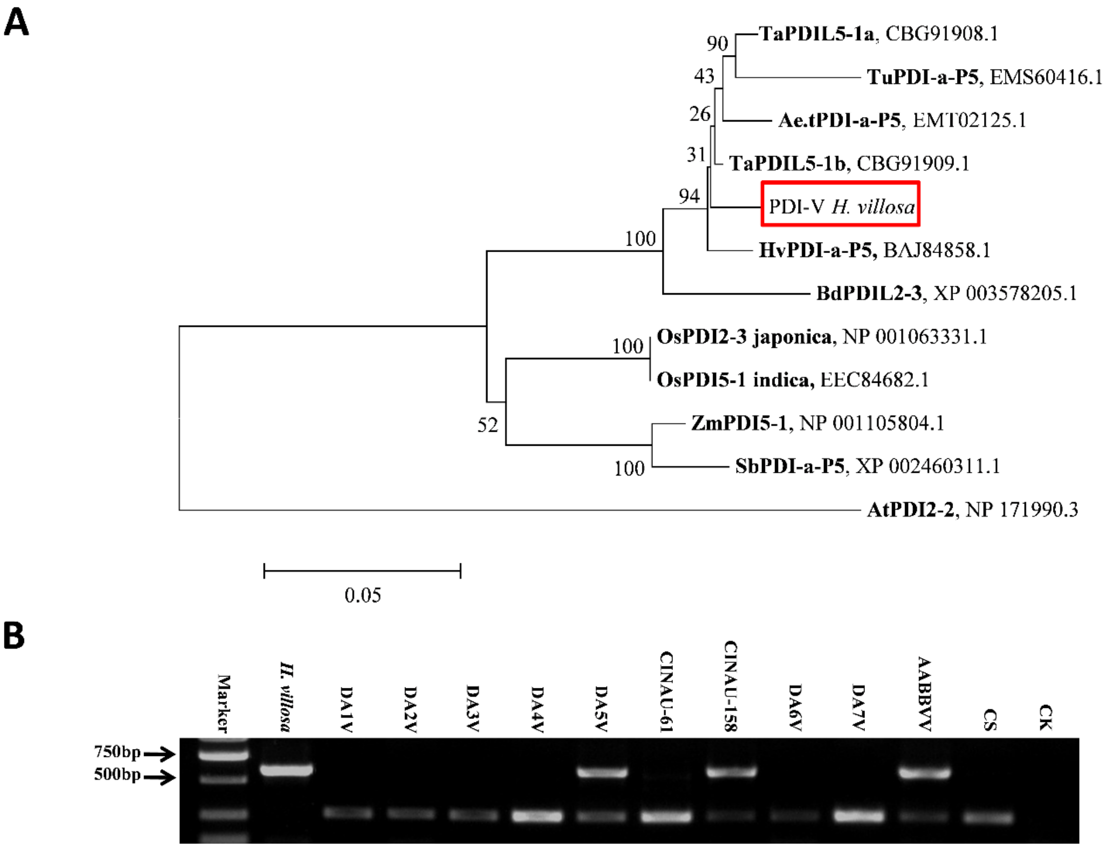

Supplementary Figure S2: (A) The phylogenetic analysis of PDI-V with other members of *Poaceae* family using MEGA 4.1 software. The tree branches ends in Gene Bank accession numbers and protein name along with species name. **(B)** Chromosomal location of *PDI-V*. Marker, DNA marker DL2000; *H. villosa*, *Haynaldia villosa*; DA1V to DA7V, *T. aestivum-H. villosa* addition lines (1V to 7V); CINAU-61, T5VS.5DL: CINAU-158, T5VL.W; AABBVV, *T. durum-H. villosa* amphiploid; CS, Chinese spring; CK, double distilled water.

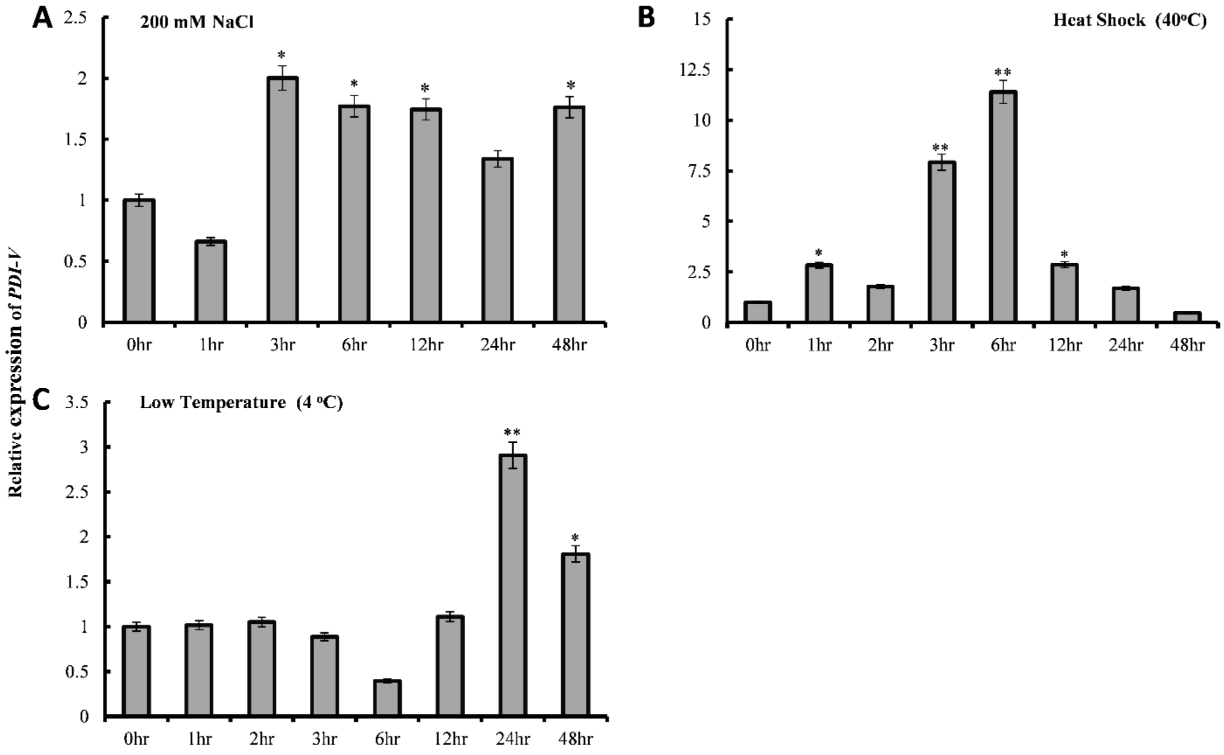

Supplementary Figure S3: Expression pattern of *PDI-V* under abiotic stresses i.e. salt (A), heat shock (B) and low temperature (C). Each result represents the average of three independent biological repeats while bars shows standard deviation. *Tubulin* gene was used as an internal control to normalize the qRT-PCR values. The asterisks indicate a significant difference ($P<0.05$) from control (0 h p.i.) using student's t-test.

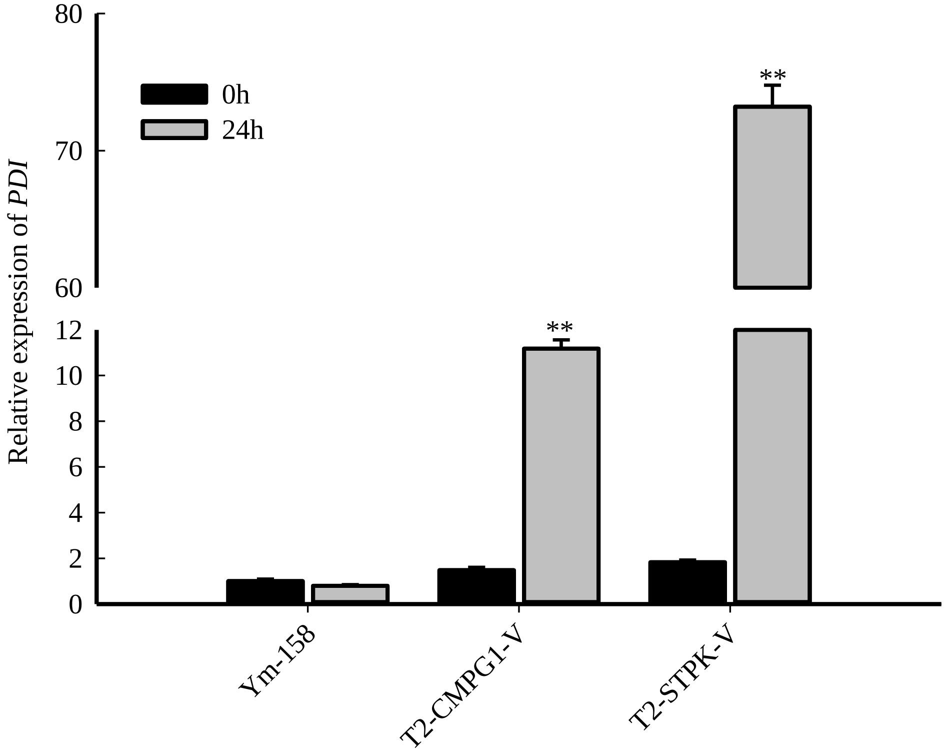

Supplementary Figure S4: Expression of *PDI* in overexpressed transgenic plants (T_2 generation) of different *H. villosa* powdery mildew resistance genes at two time points i.e. 0 h and 24 h post *Bgt* inoculation. Each point represents the mean of three replicates. Bars indicate ±SE, **p<0.01. Ym-158, wheat cultivar Yangmai 158.

Table S1: Putative CMPG1-V interaction clones and their predicted encoded proteins identified by screening of the yeast two hybrid library.

Interacting fragment	Predicted function	Fragment length (bp)
CMIN1	Retrotransposon	867
CMIN2	Farnesylated protein	630
CMIN3	Glutamyl-tRNAreductase	531
CMIN4	Chloroplast inner envelope protein	705
CMIN5	PDI-like protein	984
CMIN6	Polyubiquitin containing 7 ubiquitin monomers	678
CMIN7	Rieske Fe-S precursor protein	540
CMIN8	Pi starvation-induced protein	327
CMIN9	ARP3 actin-related protein	1056
CMIN10	Mannan endo-1, 4-beta-mannosidase	687
CMIN11	Chlorophyll a-b binding protein	792
CMIN12	EX70 superfamilyprotein	1276
CMIN13	Stress responsive protein	1226
CMIN14	ATP sulfurylase	1189
CMIN15	Ribulose-1,5-bisphosphate carboxylase/oxygenase small subunit	1250
CMIN16	ATP-dependent zinc metalloprotease	1266
CMIN17	Lectin receptor kinase	1046

Table S2: Primers used for PCR, qRT-PCR and plasmid construction

Name	Sequence (5′ to 3′)	Application
PDI-V-GST-F	CGGGATCCATGCGTCCGGCCATCCTCG	Construction of expression vector for protein *pGEX4:PDI-V*
PDI-V-GST-R	CCCCCGGGCAACTCGTCGTTGGGC	
PDI-V-CE-F	GCTCTAGAATGCGTCCGGCCATCC	Construction of BiFC vector *cYFP:PDI-V*
PDI-V-CE-R	GAAGGCCTCAACTCGTCGTTGGGC	
CMPG-NE-F	GGACTAGTATGGTCACGCCGCTGC	Construction of BiFC vector *nYFP:CMPG1-V*
CMPG-NE-R	CGGGGTACCTCAGAAGGGTCTCTTG	
PDI-V-580-F	GGACTAGTATGCGTCCGGCCATCC	Construction of subcellular location vector *pAN580:PDI-V*
PDI-V-580-R	CGGGATCCCAACTCGTCGTTGGGC	
PDI-V-40F	CCAGTTCAGTACCAAAATAATGC	Chromosomal location of *PDI-V*
PDI-V-40R	AGTAATGCACATGCGGAACA	
PDI-V-VIGS-F	CTAGCTAGCGCGCCGTGGTGCGGGCACT	Construction of VIGS vector *BSMV* γ*:PDI-V*
PDI-V-VIGS-R	CTAGCTAGCTCTCTTGCGCCTTCGTAATCC	
PDI-V-220-F	CGGGATCCATGCGTCCGGCCATCC	Construction of overexpression vector *pBI220:PDI-V and pBI220:PDI-V*^{C57A}
PDI-V-220-R	GAAGGCCTCAACTCGTCGTTGGGC	
PDI-V-a-F	CGGGATCCATGGTCCTCCAGC	Construction of overexpression vector *pBI220:PDI-V*^{a}(For amplification of nucleotide encoding amino acids from 29 to 129
PDI-V-a-R	GAAGGCCTCAGAGCAAAATTT	
PDI-V-a′-F	CGGGATCCATGGAATTGAATT	Construction of overexpression vector *pBI220:PDI-V*$^{a'}$(For amplification of nucleotide encoding amino acids from 167 to 267
PDI-V-a′-R	GAAGGCCTGTCCTTCGCGTTG	
PDI-V-b-F	CGGGATCCATGCCACCTGAAG	Construction of overexpression vector *pBI220:PDI-V*^{b}(For
PDI-V-b-R	GAAGGCCTCACCGTGGGGGCG	

		amplification of nucleotide encoding amino acids from 275 to 405
PDI-V-qPCR-F	CTGAGTTGACTAGCGCAGATGT	qRT-PCR primers for *PDI-V*
PDI-V-qPCR-R	TTCCTTCTGCCTTAGAGTCCAG	
CaMV35S-F	AGTTCATTTCATTTGGAGAGAACAC	Identification Primers for *PDI-V* and *PDI-V*^{C57A}
PDI-V-R	TTCCTTCTGCCTTAGAGTCCAG	
PR1-F	CTGTCCGACTTTGTGAGCATA	*TaNPR1* (AX049430.1)
PR1-R	CCCGCTGTCATTCTTCAGGTTG	
PR3-F	ACCTCCTTGGCGTCAGCT	*TaPR3* (CK207575)
PR3-R	TCGCACCATTATTCCCTT	
PR5-F	CAAGCAGTGGTATCAACGCAGAG	*TaPR5* (AF384146.1)
PR5-R	GTGAAGCCACAGTTGTTCTTGAT	
PR10-F	ACGGAGCGGATGTGGAAG	*TaPR10* (CV778999)
PR10-R	GCCACCTGCGACTTGAGC	
JAZ-F	CCGTAGCACGGTCTTACCAT	Unigene51573
JAZ-R	ATATGAGGCGAGCAACTTGG	
COL1-F	GCCTTGCACCCATATGTACC	Unigene22208
COL1-R	TACAGCGGCAACATCCAGTA	
Tubulin-F	AGAACACTGTTGTAAGGCTCAAC	qRT-PCR Primers for *Tubulin*
Tubulin-R	GAGCTTTACTGCCTCGAACATGG	

Table S3: *PDI-V* orthologs in different *Poaceae* species along with chromosome location, gene position and percentage homology.

**Species**	**Location**	**Position**	**% homology with *PDI-V***
Triticum aestivum (Chinese Spring, A-genome)	5AL	71824950 – 71828919	93
Triticum aestivum (Chinese Spring, B-genome)	5BL	155617833 – 155623834	92
Triticum aestivum (Chinese Spring, D-genome)	5DL	Scaffold 4603491	95
Hordeum vulgare	5HL	406411660 – 406417038	88
Oryza sativa	9Os	16930987 – 16935904	87
Brachipodium distachyon	4Bd	37609327 – 376150051	87
